# Comparison of the accuracy of monofilament testing at various points of feet in peripheral diabetic neuropathy screening

**DOI:** 10.1186/2251-6581-13-19

**Published:** 2014-01-28

**Authors:** Shahram Baraz, Kourosh Zarea, Hajie Bibi Shahbazian, Seyed Mahmoud Latifi

**Affiliations:** 1 Chronic Diseases Care Research Center, School of Nursing and Midwifery, Ahvaz Jundishapur University of Medical Sciences (AJUMS), Golestan Street, Ahvaz, Iran; 2 Diabetes Research Center, Ahvaz Jundishapur University of Medical Sciences, Ahvaz, Iran

**Keywords:** Peripheral sensory neuropathy, Diabetes, Monofilament, Screening, Nerve conduction velocity, Iran

## Abstract

**Background:**

Diabetic Peripheral Neuropathy is one of the most prevalent complications of diabetes mellitus. The development and progression of such complications are responsible for much of the morbidity and mortality. The purpose of this study was to evaluate the effectiveness of Semmes–Weinstein monofilament ten gram in 3, 4, eight and ten points in the screening of diabetic peripheral neuropathy in patients with diabetes mellitus.

**Methods:**

In a descriptive correlational design, 150 patients with diabetes mellitus were selected using convenience sampling. All patients were evaluated for sensory neuropathy using ten gram Semmes-Weinstein Monofilaments and questionnaire on neuropathy symptoms. In the next phase, nerve conduction velocity was examined. The most common subjective symptoms were paresthesia of both feet, pain in feet, burning sensation in the extremities and numbness in the extremities.

**Results:**

The results showed that the sensitivity of Monofilament in three and four points were 35.9 to 53.8 present and 38.5 to 51.3 percent respectively. Specificity of Monofilament the same points, were 73.9 to 84.7 and 73 to 87.4 percent respectively. Monofilament sensitivity at eight and ten points were 38.5 to 61.5 and 64.1 to 30.8 percent respectively. Also, specificity of the same points were 77.5 to 95.5 and 64 to 89.2 percent respectively. It was revealed that the difference sensitivity and specificity of Monofilament in three and four points with sensitivity and specificity in eight and ten point is not statistically significant.

**Conclusions:**

This study showed that Semmes-Weinstein monofilament can easily use as a simple and inexpensive device for screening. Since increasing the number of points it was not significantly difference. Therefore, we suggest that screening for diabetic peripheral neuropathy, especially in large populations to avoid wasting time on Monofilament application, areas like three or four points eight and ten points could be used.

## Introduction

Diabetes is the most common metabolic disease worldwide. IDF and WHO reported increasing incidence all around the world especially in developing countries [1]. Prevalence of diabetes mellitus (DM) in different parts of Iran is between 7.5–7.9 percent. There are more than 3 million people diagnosed by DM, which unfortunately increases three times every 15 years [2]. Diabetic neuropathy is one of the most common complications of DM which can cause motor/sensory dysfunction in diabetic patient [3]–[5]. Peripheral sensory neuropathy is one of the single strongest factors associated with the development of foot ulcers, amputations, Charcot Arthropathy, and other foot complications [6,7]. Diabetic neuropathy complications include severe pain, loss of sensation, foot ulceration and amputation, burns, infection, cellulites, sleep disorder, impaired daily functioning, mood disorders, gangrene, involvement of different systems such as cardiovascular, gastrointestinal and reproductive systems [3,8].

These complications, especially ulcer and amputation, affect the quality of patients’ life, which in turn leads to repeated hospitalizations and increased health care costs [9]. For instance, 10.15 billion dollars are annually spent for the treatment of diabetic neuropathy and its complications in the U.S. Therefore, early prevention from the complications of diabetic neuropathy is essential for the recovery of these patients [10].

Several devices are used to track the performance of protective sensation of the foot in diabetic patients. Monofilaments, along with other methods such as evaluation of pain sensation, sense of vibration, temperature sense, and deep reflexes are considered the common methods for the screening of DPN [11]. Nerve conduction test is an effective method for the diagnosis of DPN, but this method is time consuming and expensive and may not be suitable for patients’ diagnosis in outpatient departments [12]. Many other methods as an alternative method for the screening of DPN in patients with diabetes are available. Monofilaments including Semmes-Weinstein Monofilament Testing (SWMT) are among the safest and cheapest methods of DPN’s screening [6]. SWMT is so calibrated that if a force of 10 grams is applied to the extent that the monofilament is bent but the patient does not feel it, then that point is considered as insensate [13]. This is a simple test used to determine the amount of DPN and predict the possibility of foot ulceration in patients with diabetes. In this test, certain points of the feet are stimulated by putting monofilament on the skin to determine the presence of sensation. This test has high power of screening to determine the risk of foot ulceration and reduces traumatic injuries [14,15].

Nurses, by using a monofilament as screening method, also can provide the necessary training to prevent foot ulcer and amputation (such as the use of appropriate footwear, more precise control of blood sugar and fat, the monthly check of the incidence of neuropathy and increased blood sugar, and the use of walker) to those at risk of neuropathy [16,17]. In other words, teaching patients about how to use monofilament at home not only prevents from the complications, but also is a high motivational factor for patients in order to better control their blood sugar levels [18-20]. It also reduces psychological problems followed by an early diagnosis of DPN in susceptible patients alongside increasing in patients’ quality of life [15,21].

Clinical care guidelines have recommended that annual screening for peripheral neuropathy occur in all patients with diabetes, as part of the routine evaluation of patients in terms of complications [22]. The sensitivity of the SWMT used by Miranda-Palma to track the reduction of sensation in three points of the sole was reported to be between 65% to 86% [23]. Nather et al. (2008) compared the incidence of neuropathy diagnosed by three devices: pin-prick, SWMT, and neurometer. It was shown that the rate of sensory neuropathy diagnosed by the pin-prick and neurometer tests was significantly higher than that of SWMT [24]. The SWMT is so calibrated that a force of 10 grams is applied to the extent that the monofilament is bent, but if the patient does not feel it, that point is considered insensate [15].

In the international study this test was conducted on 10 and 15 points, and the results have been different. Since in the most studies, a 10 g monofilament has been used often for screening, and no study still has been conducted for comparing 10 g monofilament in different points carefully, this study was designed with the following objectives: The first aim of our study was to answer the question that whether increasing the number of points to use in screening monofilament neuropathy can be accompanied with high sensitivity? The second objective was to compare the sensitivity and specificity of monofilament with the Modified neuropathy disability score.

## Methods

A quasi-experimental design was used to conduct this study. This research was conducted from 2010 to 2011 with 150 patients suffering from type II, DM referred to a Diabetic Clinic affiliated to a teaching hospital in Ahvaz, Iran.

Using a statistical sampling formula with *α* = 0.05, *β* = 0.2, *p*_1_ = 0.86, *p*_2_ = 0.96 adopted from other studies [23] the number of samples was 150 people

n=z1−α2+z1−β2p11−p1+p21−p2p1−p22=148≅150

The patients with type II, DM with the following criteria entered the study: (a) having no diabetic foot ulcer; (b) having a stable clinical condition; (c) having no psychological problem, (d) having no history of brain stroke, and neuropathy due to other reasons such as hereditary-acquired neuropathy or the Guillain-Barre Syndrome; (e) having no callous or any other complications in feet; (f) being able to communicate verbally; and (g) Being interested in participating in this study.

### Ethical considerations

After the approval of the study at the Ethics Committee of Jundishapur Medical Sciences University (No.3040), Ahvaz, Iran, informed consent was taken from the patients for participating in the study. Also, the patients received enough information about the safety of this method and their freedom for entering into or exiting the study.

### Instrument

#### Questionnaire on neuropathy symptoms

In order to examine the mental symptoms of neuropathy, The following 13 questions were Prepared: (1) pain in hands; (2) pain in feet; (3) numbness at terminal points; (4) paraesthesia of both feet; (5) burning sensation in the extremities; (6) cold sensation in the extremities; (7) constipation; (8) diarrhea; (9) muscular cramps; (10) fainting; (11) dizziness; (12) problems in urinating; (13) ulcers at feet. These symptoms were classified to the following 4 categories: asymptomatic, mild, moderate, and sever. The patients were accordingly divided to two groups of asymptomatic and symptomatic (the total of mild, moderate, and sever) [25].

#### Semmes-Weinstein Monofilament Testing (SWMT)

In this study, we used Semmes-Weinstein monofilament (North Coast Medical, Inc., San Jose, CA) with a reproducible buckling stress 10 grams. Monofilament testing was performed on both feet of the patients by the first author, who had received the necessary training on the use of monofilament. The test at 3, 4, 8, and 10 points of the feet were evaluated. Three points in each foot were the great toe, the plantar aspect of the first metatarsal head; and the plantar aspect of the fifth metatarsal head. Four points in each foot, including: the plantar surface of hallux, and the first, third, and fifth metatarsal heads. Eight points in each foot, including: the dorsal aspect of the first, second, third, fourth, and fifth digits; the dorsal aspect of medial, central, and lateral aspect of mid foot and ten points in each foot, including: nine plantar sites (distal great toe, third toe, and fifth toe; first, third, and fifth metatarsal heads; medial foot, lateral foot, and heal) and one dorsal site.

### Procedure

The monofilament was accidentally placed on palm of the patient’s hand while his/her eyes were closed and each patient was asked the following questions:

•Do you feel the monofilament on the palm?

•Which part of your hand is in contact with the monofilament?

After it was ensured that the patient understood how to cooperate and how to answer questions, the monofilaments on the bottom of both feet of the patient were placed, while the patient’s eyes were closed. The monofilament was placed on the portion of the patient’s skin, which had no callus, and was pressed as far as the monofilament could be bent. The patient was asked whether he/she felt something on the sole and on which foot he/she felt the monofilament. In each point, the test was repeated for three times. If the patient answered incorrectly two or more times in that point, it was recorded as a positive symptom of neuropathy [25]. The duration of conducting the test on both feet was for 5 to 10 minutes.

#### Modified neuropathy disability score (NDS)

This score (maximum 10) is derived from abnormalities of pain sensation using a Pin-Prick, vibration sensation using a 128 Hz tuning fork, dorsal temperature sensation using warm and cold rods and Achilles tendon reflexes using a tendon hammer [26,27].

### Examination of pain sensation

Pin prick test was used for this purpose. First, the patient was asked whether he/she felt needles sensation or numbness in the hands or feet. None of the patients experienced sensory impairment in their hands. Then, the test was performed with the pin using a standard procedure for all patients by the second author. In this procedure, while the patient’s eyes were opened, the pin prick was vertically pressed on the skin behind the forearm of the patient until the patient began to express pain. The same test was then repeated with the same amount of pressure on the dorsal surface of the hand to cause pain. Then parts of the toes, forefoot, midfoot, hind foot were tested with the same pin and with the same amount of pressure and the patients were asked whether the pain in the feet is more (hyperesthesia)/less (hypoesthesia) than or similar to the pain in the hands.

Then in the next step, while the patient’s eyes were closed, the test was performed with the above procedure and the patient was asked to raise his/her right hand when he felt pain. The locations that had sensory impairment (either as hyperesthesia or as hypoesthesia) were noted as positive sites. There are two types of sensory disturbances indicating the presence of neuropathy. For each patient, a pin with the same type was used [24].

### Testing of the temperature sense

Hot and cold water pipes were used on the patient’s thumb. Temperature of the cold water pipe was not less than 25°C, and temperature of the hot water pipe was not more than 45°C, because otherwise it would cause pain [24].

### Examination of the sense of vibration

A 128 Hz tuning fork was used. First, the patient was taught, by placing the vibrating tuning fork on the forehead or sternum, that understanding the sense of shaking or vibration is intended, not just the tuning fork’s contact with the body. After we realized that the patient understood what we meant, we vibrated the tuning fork with the stroke of the palm of the hand, and it was put on the bony prominence on the back of the thumb. The patient was asked to report the perception of both the start of the vibration sensation and cessation of vibration on dampening [24].

### Achilles reflex testing

Achilles reflex was evaluated on the condition that the patient was bent on the stomach and his/her knees were bent at 90 degrees. We stroked Achilles tendon, and saw the patient’s response. Normal response is as a plantar flexion and abnormal responses were noted as either decreased reflexes or absent reflexes [24].

For one foot, each sensory test scored zero for normal sensation or one for abnormal sensation: ankle reflex scored zero if present, one if present with reinforcement or two if absent. Pain and temperature sensation were assessed on the dorsal surface of the great toe after the stimuli were demonstrated at a proximal, normal site. Vibration perception was assessed using the 128 Hz tuning fork over the apex of the hallux. The Achilles tendon reflex was assessed with the patient supine on a couch. The maximum score for the two feet is 10, with a score of ≥6 indicating moderate to severe neuropathy [26,28].

### Diagnostic criterion for DPN

In order to diagnose DNP, the nerve conduction velocity (standard test) was also measured. The technician for measuring the nerve conduction velocity was quite blind about the clinical Testing. To identify the sensitivity and specificity of the SWMT, the results of the monofilament Testing were compared with the nerve conduction velocity as the gold test. The neural conduction tests are recommended by other studies as the gold test for assessing and validating screening tests for diabetic neuropathy [29]–[31].

### Data analysis

The data analysis was performed using the SPSS software Ver.16. The Sensitivity and specificity of the SWMT were measured. The data were presented as means and standard deviations, and percentiles.

## Results

From among the 150 patients participating in the study, there were 47 males (31.3%) and 103 females (68.7%). The average age of the patients was 55.71 ± 8.95 years and the mean of disease duration was 7.7 ± 6.1 years. The mean fasting blood glucose, the mean HbA1c, the mean cholesterol, the mean triglycerides, the mean HDL Blood, the mean LDL Blood level, the mean BMI and the mean weight in patients were 169.92 ± 72.66, 8.26 ± 3.96, 182.31 ± 44.10, 127.49 ± 54.87, 47.21 ± 12.24, 100.21 ± 34.09, 27.45 ± 4.85, and 55.35 ± 8.99, respectively. All patients were under treatment with oral medication. Drug regimen for most patients was *glibenclamide* and *metformin* (Table 1).

**Table 1:** Demographics variables in the patients

**Demographic variables**	**Mean ± SD or n (%)**
Age	8.95 ± 55.71
Sex: Male	47(31.3)
Female	103(68.7)
Duration of disease	6.1 ±7.7
FBS	72.66 ±169.92
HbA1_c_	3.96 ± 8.26
CHLO	44.10 ± 182.31
TG	54.87 ± 127.49
HDL	12.24 ± 47.21
LDL	34.09 ± 100.21
BMI	4.85 ± 27.45
Weight	8.99 ± 55.35

The results showed that the majority of patients (75. 34%) have had HbA1c levels greater than 7%. It is shown that these patients have had a poor control over their diabetes. Among the clinical manifestations of neuropathy, paresthesia (72%), pain at feet (71.3%), feeling of pins and needles at feet tips (71.3%), numbness at terminals (63.3%), pain in hands (61.3%), and coldness at feet (50.7%) had the highest prevalence among the patients. Totally, through the examination of symptoms, 93 patients were healthy and without neuropathy and 57 individuals (38%) had neuropathy.

Testing with the 10 g monofilament was assessed according to different cut-off points for positivity (Table 2). (a) From 6 points, at least one point as a local insensate has been reported by the patient, (b) From 6 points, at least two points as a local insensate have been reported by the patient and (c) From 6 points, at least 3 points as a local insensate have been reported by the patients.

**Table 2:** Sensitivity and specificity of the 10 g monofilament according to the number of insensate sites

**Testing site**	**Sensitivity**	**Specificity**
Monofilament ≥1/8^*^	51.3	73
Monofilament ≥ 2/8^†^	46.2	74.8
Monofilament ≥4/8^‡^	38.5	87.4

*Minimum of 8 point 1 point reported by patients as a numbness site.

†Minimum of 8 point 2 point reported by patients as numbness site.

‡Minimum of 8 point 4 point reported by patients as numbness site.

The procedure for 4 points, 8 points, and 10 points were used in each plantar foot. So that a minimum one points out of 8 points, at least two points out of 8 points, and 4 points out of 8 points at numb have been reported. At least, one point of 16 points, two points out of 16 points, eight points out of 16 points at numb have been reported. At least, one point out of 20 points, two points out of 20 points, 10 points out of 20 points at numb have been reported (Tables 3, 4 and 5).

**Table 3:** Sensitivity and specificity of the 10 g monofilament according to the number of insensate sites

**Testing site**	**Sensitivity**	**Specificity**
Monofilament ≥1/6^*^	53.8	73.9
Monofilament ≥ 2/6^†^	43.6	79.3
Monofilament ≥3/6^‡^	35.9	84.7

*Minimum of 6 point 1 point reported by patients as a numbness site.

†Minimum of 6 point 2 point reported by patients as numbness site.

‡Minimum of 6 point 3 point reported by patients as numbness site.

**Table 4:** Sensitivity and specificity of the 10 g monofilament according to the number of insensate sites

**Testing site**	**Sensitivity**	**Specificity**
Monofilament ≥1/20^*^	64.1	64
Monofilament ≥ 2/20^†^	61.5	64
Monofilament ≥10/20^‡^	30.8	89.2

*Minimum of 20 point 1 point reported by patients as a numbness site.

†Minimum of 20 point 2 point reported by patients as numbness site.

‡Minimum of 20 point 10 point reported by patients as numbness site.

**Table 5:** Sensitivity and specificity of the 10 g monofilament according to the number of insensate sites in 16points according to insensate sites

**Testing site**	**Sensitivity**	**Specificity**
Monofilament ≥1/16^*^	61.5	77.5
Monofilament ≥ 2/16^†^	59	79.3
Monofilament ≥8/16^‡^	38.5	95.5

*Minimum of 16 point 1 point reported by patients as a numbness site.

^†^Minimum of 16 point 2 point reported by patients as numbness site.

^‡^Minimum of 16 point 8 point reported by patients as numbness site.

We also compared the results of monofilament with the improved sensitivity and specificity of the improved neuropathy criterion and the tuning fork tests (Table 6). Patients who acquired scores greater than, or equal to, 6 of the improved neuropathy criterion, were defined as patients with neuropathy symptoms [15,30]. The sensitivity and specificity of these two criteria were obtained 91% and 94.9% respectively.

**Table 6:** Sensitivity and specificity according to methodologies used to assess insensitivity

**Test**	**Sensitivity**	**Specificity**
Monofilament ≥1/6	53.8	73.9
Monofilament ≥1/8	51.3	73
Monofilament ≥1/16	61.5	77.5
Monofilament ≥1/20	64.1	64
NDS	94.9	91
128 Hz tuning fork^a^	84.6	43.2

^a^≥1 of the two sites tested was considered positive.

Results of the improved neuropathy criterion showed that of the 150 patients with neuropathy participating in the study, 138 patients have acquired scores equal to or greater than 6, and it shows that according to this criterion, 92% of our patients had been moderate to severe symptoms of neuropathy.

Moreover, the tuning fork testing that is part of NDS criterion was evaluated separately. Thus, the lack of the tuning fork vibration sense on one of the big toes was used as a positive point. Sensitivity and specificity of tuning fork was alone at these points 84.6% and 43.2% respectively. The sensitivity and specificity of both tests were greater than monofilament.

## Discussion

The use of this test in our study showed that when factors such as cost and ease of use are concerned, then 10-g Semmes-Weinstein monofilament are effective for detection and screening of the foot protective sense reduction about the diabetic foot. In this study, we applied Semmes-Weinstein monofilament in 3, 4, 8 and 10 points per the sole of the foot. If we look at the results of monofilament in these points, we see that the sensitivity of monofilaments was in the range of 30.8%– 64.1%. This relatively low sensitivity implies that these abnormal areas and the numb points only can be diagnosed in very severe neuropathies. Also, according to the obtained results, we see that sensitivity and specificity of monofilament in 3points and 4points have not statistically significant difference with sensitivity and specificity in the 8 points and 10 points. In other words, with increasing the number of studied points, the monofilament sensitivity has not been increased. Therefore, we recommend that physicians or nurses use from fewer points like 3 points and 4points to screen the patients with diabetes, and do not spend their time to use monofilament in more points.

In this study, the sensitivity of monofilament in 3 points and specificity of this test were (38.5%–51.3%) and (73%–87.4%) respectively. Kamei in his study, reported sensitivity and sensitivity of 10-g Semmes-Weinstein monofilament in 3 points, 5%–22.5% and 88.1%–97.6% respectively [25] that compared with our study, the sensitivity of monofilament was less, but its specificity was higher; this can be on a number of samples, sample type and other characteristics of patients and limitations of each study.

Moreover, in this study, the sensitivity and specificity of monofilament in the 4 points were 38.5%–51.3% and 73%–87.4% respectively. Miranda-Palma et al., reported the range of sensitivity and specificity of Semmes-Weinstein 10 g monofilament at four sites, from 65% to 86%, and 58% to 71%, respectively [23]. In this study, the sensitivity and specificity of monofilament in 8 points were 38.5–61.5 and 77.5%–95.5 respectively. Also the sensitivity and specificity of monofilament at 10 points were 30.8%–64.1% and 64%–89.2% respectively. Mason and colleagues reported sensitivity and specificity of monofilament at 10 points, respectively 92.1%, and 100% [30].

Although the use of monofilament has been started since 1995, but the number of points that must be considered is still under study. In our study, the prevalence of diabetic neuropathy on the basis of a positive test 10–g Semmes-Weinstein monofilament is the 9.33%–24% [32]. This finding is different from other studies, including in Forouzandeh’s study, prevalence of diabetic neuropathy based on to be a positive monofilament test was 23.9%, which this case could be due to the number of samples involved, the type of sampling, the number of points to be considered [29].

Nevertheless, the aim of our study was the use of monofilament as a screening measure of peripheral neuropathy, while they used monofilament to track down and detect the numb foot to prevent lower limb amputation. Furthermore, their study differs from ours, because they compared the sensitivity and specificity of 10-g monofilament with vibration test as the gold test while we used nerve conduction tests (NCV) as the gold test.

The above examples demonstrate that subtle changes in abnormal response to monofilament can cause differences in the prevalence of numbness in feet between 3.4%–29%. On the other hand, for simple things like monofilament, small changes in the force imposed to monofilament can lead to misleading results and unusual effects. For this reason, there is a disagreement for its use in distinctive countries which it can lead to an ambiguity in epidemiological researches and to estimate the need in the health care [3,15,31,33]. More research is needed to investigate the environmental condition’s effects such as temperature, and moisture that can affect the force required to bend the monofilament (which is usually 10 grams) [34].

In this study, we used a questionnaire for subjective signs in order to investigate the neuropathy. Results of Testing of the signs showed that 93 persons were healthy and without signs of neuropathy, and 57 persons (38%) were with neuropathy. Pain and tingling sensation had the most prevalence among the clinical signs of paresthesia. In 78.7% of the patients, knee reflexes diminished or disappeared; and in 64% of patients, vibration sense was impaired. Our content can be also confirmed by Kamei’ study, where 51.2% of patients suffered from the pain, numbness, and paresthesia in both legs. In 56.1% of the patients, knee reflexes were reduced or eliminated; and vibration test in 36.6% of the patients was impaired. In this same study, the majority of patients had muscle cramps, numbness in the extremities, and the moderate to severe pain [25].

Monofilament test cannot be favorably compared with other methods. The results of a comparison between monofilament tests with standard NDS showed that the sensitivity and specificity of NDS criterion are high. While the results of Miranda Palma’s study showed that the sensitivity of monofilament is higher than NDS, but its specificity was lower than in the standard [24]. Therefore, the different results obtained certainly cannot say which of these methods are superior to both It must be considered that if these methods can be used together, then more accurate results can be achieved.

Although other studies have used the NDS as the accurate and useful method for investigating the environmental sense, but reasons to use the monofilament method are to be easy, to be available, and to be cheap [3,9,24,32]. It should be noted that the time required for this test is pretty short. However, NDS require more time and equipment and skills. In other words, employing monofilament by a doctor or nurse or even the patient is a lot easier. So that using monofilament, and screening, the patients can prevent the undesirable complications such as foot ulcers and amputation. With the help of this test, we can identify these individuals susceptible to, and at risk, and present the necessary instructions to them for preventing the foot ulcers and amputation such as the use of proper footwear, more accurate control of blood sugar and blood fat. Blood sugar checked monthly incidence of neuropathy and increased use of Walker and others.

Associated with glycemic control, HbA1c and fasting blood-glucose levels in this study showed that most patients have poor blood-glucose control and were not able to maintain normal blood sugar range themselves. Therefore, this study can provide evidence for physicians, nurses, and healthcare policy makers, so they can make changes in the current health system, including to provide the necessary and sufficient training to patients, and to follow the screening of the patients more seriously in both hospital wards and outpatient clinics.

Moreover, we investigated screening for diabetic peripheral neuropathy in our study, and concluded that none of the patients involved in the study were not studied in terms of diabetic peripheral neuropathy and its complications. In other words, the present study is the first study to screen the patient with diabetes. These findings show that the screening of diabetic peripheral neuropathy is poor in Ahvaz that should be improved.

## Conclusions

Overall, by increasing the number of points for neuropathy screening, the sensitivity monofilament did not increase. In other words, the sensitivity of monofilament in 3 and 4 points is almost similar to it in 8 and 10 points.

The use of monofilament solely or in combination with NDS or other reflex tests for neuropathy screening method is an easy and accessible method; and by early detection, it can prevent complications that include leg ulcers and amputation in patients with diabetes. The Testing also seems to be important in this context, and it cannot be released from it along with taking a profile.

## Competing interests

The authors declare that they have no competing interests.

## Authors’ contributions

SHB: Study design, data collection/analysis, drafting of manuscript, supervision. KZ: administrative/technical/material support, critical revisions for important intellectual content. HBSH: administrative/technical/material support, supervision. ML: Study conception, data analysis, and supervision. All authors read and approved the final manuscript.
